# QFD-Based Research on Sustainable User Experience Optimization Design of Smart Home Products for the Elderly: A Case Study of Smart Refrigerators

**DOI:** 10.3390/ijerph192113742

**Published:** 2022-10-22

**Authors:** Yongchuan Li, Raja Ariffin Raja Ghazilla, Salwa Hanim Abdul-Rashid

**Affiliations:** 1 Department of Mechanical Engineering, Faculty of Engineering, Universiti Malaya, Kuala Lumpur 50603, Malaysia; 2 Centre for Sustainable and Smart Manufacturing, Faculty of Engineering, Universiti Malaya, Kuala Lumpur 50603, Malaysia

**Keywords:** QFD, smart refrigerator for the elderly, user experience, Kano model, PUGH concept selection

## Abstract

In the current situation of global aging, the current market shortage of age-appropriate smart home products and the recent epidemic have led to greater isolation of the elderly, seriously affecting their physical and mental health. In order to optimize the sustainable user experience of the elderly when using smart home products, this paper proposes a research method based on Quality Function Deployment (QFD) for the optimal design of user experience of smart home products for the elderly, taking the design of age-appropriate home smart refrigerators as an example. Firstly, based on the results of market research and user interviews, the requirements of smart refrigerators for the elderly are screened and integrated, and the Kano model is used to prioritize these needs, resulting in the identification of important features needed in smart refrigerators for the elderly. Secondly, based on QFD, user requirements are transformed into design requirements, and a quality house model is established to ascertain the degree of importance of each design requirement through user ratings so as to obtain the key requirements as the theoretical basis for the solution design. Finally, optional solutions are generated for concept evaluation based on PUGH concept selection, comparing the advantages and disadvantages of the solutions and recombining them into an evaluation to determine the best solution. The quantitative evaluation of the four solutions reveals that Solution A has the highest score of 117.358, followed by Solution D with 113.259, Solution B with 96.415, and Solution C with 85.511, which is the lowest. The scoring allows the best design solution to be selected and applied to product development. The results show that the introduction of the Kano model and PUGH concept selection into QFD can be effectively used as a research method for optimizing the user experience of smart refrigerators for the elderly, and a corresponding design strategy for sustainable user experience optimization is proposed. The method and strategy provide guidance for the innovative design of new smart home products.

## 1. Introduction

With the rapid development of smart technologies, smart home companies around the world are seeking innovative ways to attract older consumers to use their smart home products. The firms aims are to broaden their revenue streams, drive industry innovation, and provide a more comfortable and healthy lifestyle for seniors, enhancing their independence and quality of life. This is in line with the third and ninth UN Sustainable Development Goals, “Good Health and Well-being” and “Industry, Innovation and Infrastructure,” respectively, which promote healthy lifestyles and the well-being of people of all ages, together with the building of risk-resilient infrastructure, promoting inclusive and sustainable industry, and fostering innovation [1].

Despite the fact that young people were not overwhelmingly impacted by their physical separation from social life during the COVID-19 outbreak, the elderly were more clearly isolated, owing to a lack of intelligent equipment infrastructure, which exacerbated their physical and mental health issues [2,3]. As the size of the elderly population continues to grow, it has come to constitute a large consumer group; the elderly consumer group has received increasing attention from the design field. At the same time, the trend of intelligent home products has become the new normal in the homes industry, but most smart home products in the current market are mainly designed for young people, ignoring the needs of elderly groups in the home. Therefore, it has become an urgent problem to fully explore the real emotional and other needs of the elderly, and to design and develop smart home products centered on elderly users. Among them, the conversion of household refrigerators from functional to intelligent refrigerators is a typical case of home intelligence, and the product can effectively meet the needs of the elderly as regards their technological life while improving their quality of life. With age, physiological and psychological problems, such as the sensations and perceptions, will directly affect the use of smart refrigerators, so the research on the user experience of smart refrigerators for the elderly is more and more important.

The research related to smart refrigerators focuses on technological innovation and product intelligence modification; for example, in a study of smart refrigerator design using IoT technology, Gupta et al. [4] and Sharma et al. [5] proposed the design of a smart refrigerator using Internet of Things (IoT), which will enable the monitoring of the food in the refrigerator. This study defines the working principle of a smart refrigerator, which can determine by itself when the food needs to be replenished, and also the type or quantity of food stored in the refrigerator. This research perspective is different from that of Wu et al. [6] and Dekoninck et al. [7], which describes the overall design of a low-cost smart refrigerator built using IoT-related technologies. However, at this stage, there are few studies focused on optimizing the user experience of smart refrigerators for elderly users.

QFD is a method employed to translate customer requirements into design goals and the key points needed to ensure quality at the production stage [8], and it obtains these relevant requirements through qualitative and quantitative analyses. However, the needs of the elderly in smart refrigerator user experience are complex, and using only a single research method leads to a single perspective, thus limiting the breadth and depth of the research. The Kano model is used to effectively incorporate customer preferences into product design while achieving an optimal trade-off between customer satisfaction and producer capabilities [9]. PUGH concept selection is used during concept screening and concept scoring—PUGH concept selection is a method used to help designers optimize design solutions and identify the best solution for improving user experience satisfaction [10]. However, few studies have combined the Kano model and PUGH concept selection in QFD as a design method to optimize the user experience of smart refrigerators for the elderly.

Therefore, this study introduces the Kano model and PUGH concept selection into QFD, and proposes a QFD-based design method used to provide designers with objective and effective design solutions to solve the problem of product user experience satisfaction in the elderly when using smart refrigerators.

This paper is organized into eight sections. The Section 1 introduces the background and the objectives of the study. The Section 2 comprises the literature review and the theoretical background of this study. In Section 3, the research methodology is elaborated. A representative case study is presented in Section 4. Section 5 discusses the case study and the proposed design strategies for user experience optimization. The implications and limitations of this study, and the conclusions and directions for future research, are given in Section 6, Section 7 and Section 8.

## 2. Theoretical Background and Literature Review

### 2.1. Quality Function Deployment

**Figure 1:**
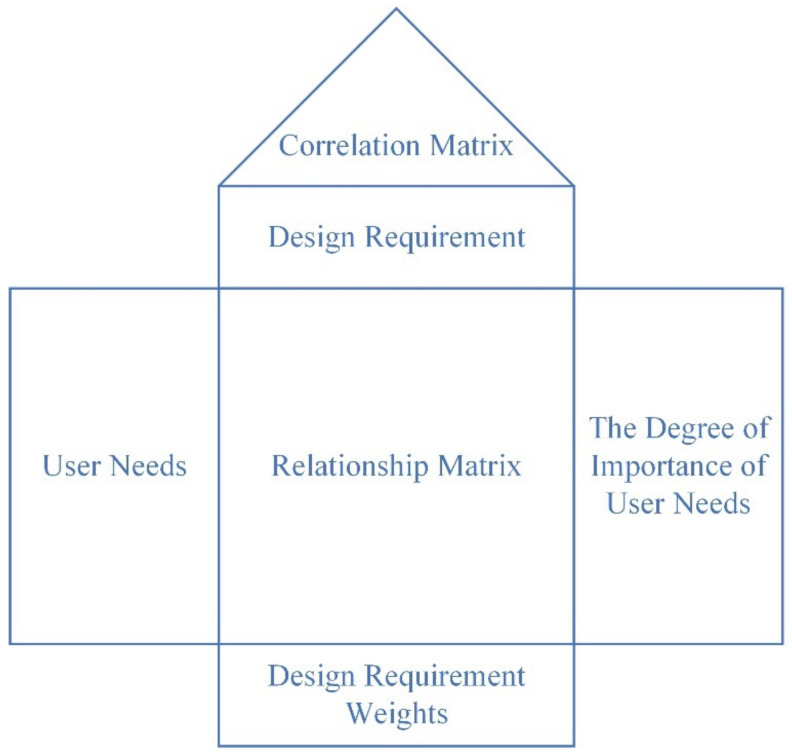
QFD house of quality.

Quality Function Deployment (QFD) is a powerful tool used in product development for obtaining and improving information on customer requirements, and for communicating the voice of the customer in design engineering to enhance user satisfaction. It is also the ultimate tool for increasing time and resource savings from the design to production planning stage [11]. The basic principle of QFD is to use the quality house matrix (see Figure 1) to identify, through data analysis and processing, the core design requirements that maximize customer satisfaction. In Japan, Mitsubishi Heavy Industries was the first to use the QFD methodology in ship design, and it achieved significant results, not only in terms of lowering R&D costs, but also in reducing the failure rate of the product design [12]. Since then, QFD theory has been adopted by large number of companies in various industries, both in Japan and worldwide, and has achieved wide success.

In the manufacturing and information technology industries, Ali et al. [13] have used the QFD method to improve the layout design of handicraft facilities so that the desired solution that meets workers’ expectations as regards the facility layout could be obtained at the design stage, before the actual layout implementation. Huang and Xu [14] used the QFD method in evaluating the master template of process protocols by examining the quality of the product in different processes, and the quality of the product to be achieved. Rao [15] used the QFD method to subdivide the e-Government online service quality characteristics and rank the demand for each, which provides a new means of optimizing the e-Government online service quality. In product service system concept design (PSS), Sousa-Zomer and Miguel [16] used QFD to translate requirements into engineering metrics for products and services to evaluate and select PSS design. Bajčetić et al. [17] used QFD to identify the needs and requirements of urban bus users to improve the quality of urban public passenger transport services. QFD has also been applied to design the service business model; Wu [18] applied QFD to conduct a study on how to improve service innovation, optimize service quality, and improve user satisfaction in the retail industry.

Among the applications in business and management science, Singh and Rawani [19] proposed an industry-oriented quality management approach with the help of an integrated QFD-TOPSIS approach. The methodology was applied to a case study of engineering education in India to improve the quality of engineering education. In addition to the field of education, the “performance management house” has been proposed as a reference source in an organization’s development of a performance management system that helps the human resource department to identify strategic opportunities and set goals to promote stakeholder satisfaction, employee engagement, and continuous improvement in the context of QFD from the point of view of business management [20].

### 2.2. Kano Model

The Kano model is a methodological tool proposed by Japanese professor Noriaki Kano to develop a technique for identifying user needs and expectations through preference classification, which helps designers to identify user needs and exceed user expectations [21]. Due to its practicality, the model was soon widely used in various industries. Depending on the characteristics of each industry, the Kano model is often used in combination with other research methods to better meet the needs of the respective industries. A considerable number of studies have combined the Kano model with QFD methods as a theoretical basis to optimize design solutions. In Sireli et al.’s [22] study on Multiple Product Design, it was noted that the combined Kano and QFD approach can prioritize design requirements based on their importance values, which can help in assessing the impact of design requirement features on the meeting of customer demand expectations. Tontini [23] combined the Kano model with the QFD approach to prioritize corporate product features, thus providing a more effective means of corporate product development; Chen et al. [24] combined the Kano model with Taguchi’s experimental design to rank product design parameters that include customer satisfaction as a way to derive the best combination of product parameters. Tian et al. [25] combined the Kano model with perceptual engineering. They distinguished three areas of modeling perceptual needs in respect of bicycles—basic, expectation, and excitement—and used them to provide directions for bicycle modeling design. Chang et al. [26] used the Kano model to rank the importance of user needs for modeling quality characteristics of UAVs, and then provided a basis and reference for UAV modeling design.

### 2.3. PUGH Concept Selection

The PUGH concept selection method is a method developed by Pugh in the 1980s of forming a concept screening matrix for evaluating concepts based on the principles of product concept selection [10]. There has often been a lack of rigor in the selection of solutions due to the involvement of large amounts of personal subjective emotion in the selection of design solutions [27]. Therefore, using the PUGH concept selection method can help establish an effective quantitative decision system for the selection of solutions, and allow designers to obtain the best of multiple solutions. Li et al. [28] combined PUGH, Maslow’s hierarchy of needs theory, etc., to determine the PUGH judgment criteria, and eventually verified its effectiveness in the selection of specific of design applications and solutions. Cao et al. [29] used TRIZ theory and evolutionary tree theory to deduce and form multiple design solutions, and they used PUGH to screen and evaluate them to select a product design solution that meets the requirements. In the early 1990s, American scholars considered Pugh in a study of various techniques, such as QFD, and introduced the concept of quality function configuration as well, which led to in-depth research work [30]. For example, in Pandit’s [31] study, the PUGH concept was selected to be applied to QFD to help select an appropriate trolley design solution.

### 2.4. Smart Home Product Design for the Elderly

The research on the design of smart home products for the elderly mainly focuses on the development and implementation of intelligent technologies, interactive interfaces, and the adoption of technology by the old. Tapia et al. [32] developed ubiquitous sensor applications that may be applied in homes, proposing that small, basic sensor facilities can make the lives of the elderly more convenient. Portet et al. [33] studied in depth a voice-based recognition system for smart homes for the elderly, arguing that through voice remote control and communication, easier communication between the elderly and their families can be achieved, and home intelligence can be enhanced, making the lives of the elderly more convenient. Pérez-Espinosa et al. [34] suggested a technique for measuring the quality of speech-based interactions based on the automated detection of audio paralinguistic events. This approach aids in the automated estimation of the quality of user-perceived interactions, and it facilitates the development of speech-based systems that modify and tailor the content and style of user interaction. Epelde et al. [35] developed a novel method for integrating the interactive services offered by ambient intelligence settings with television sets, allowing for the integration of various television set configurations and ensuring the development of generic solutions. Using customized graphical user interfaces, navigation menus, and multimodal interactions, this method has been implemented as a multimodal/multipurpose natural human–machine interface for the elderly. Several additional studies have examined the willingness of older persons (and the attributes that contribute to it) to adopt currently available smart home technology that may contribute to a safe and active lifestyle within the house [36,37].

### 2.5. User Experience Design for Seniors

Research on user experience design for the elderly has concentrated on three levels: firstly, in terms of research on older users, Dodd et al. [38], for instance, systematically analyzed the user experience differences between elderly users and general younger users during interface interaction by conducting a literature review. They noted that elderly users may have decreased functional ability, weak perception of product or system experience, and cognitive decline, all of which impact user performance and experience, and proposed corresponding user interface development solutions for the identified problems. Secondly, research on the user experience of the elderly has employed qualitative and quantitative approaches, such as ethnographic methodologies, questionnaires, etc., to identify the obstacles that arise in their use of equipment and the actual demands of users. Cheng and Li [39] explored the user experience process of electronic goods for the elderly by combining ethnography, interaction experience design, and explicit design with tacit knowledge. They concluded that the design of electronic products for the elderly should reflect their actual requirements. Hashizume et al. [40] conducted a study on the user experience of smartphone use by older adults through questionnaires and field interviews to investigate the motivations, barriers, and differences in the use experience of older adults compared to younger populations at a broader activity level. Thirdly, in terms of the user experience assessment of older adults, Zhang [41] developed a user experience assessment system for smartphone use in the prime-age population based on the Perceived Usability and Acceptability Integration Model and used a comprehensive analysis mode to clarify smartphone design features and design a smartphone suitable for this population group.

### 2.6. Research Gap

After reviewing the previous literature, we can identify the following research gaps. First, the extant literature suggests that fewer practical smart home products are designed for the elderly, and there is a lack of in-depth and systematic theoretical analysis and methodological exploration from the perspective of user experience. Second, although some scholars have paid attention to smart home product design for the elderly, they mainly focus on the technical level, and few pay attention to the optimal design of user experience for the elderly, especially for those who rely on smart products in their daily lives. Third, the approach of introducing the Kano model and PUGH concept selection into QFD not only helps to accurately identify the real user needs, but it also ensures that the user needs are communicated and reflected in the product design in a timely and effective manner, and that the best design solution is derived from the various possible design options. Reviewed literatures suggest that this combination of methods has never been applied in the field of user experience enhancement for smart home products for the elderly. Therefore, it is critical to comprehensively address these barriers and gaps in order to reduce the negative physical and psychological impacts of the epidemic on the elderly.

## 3. Methods

**Figure 2:**
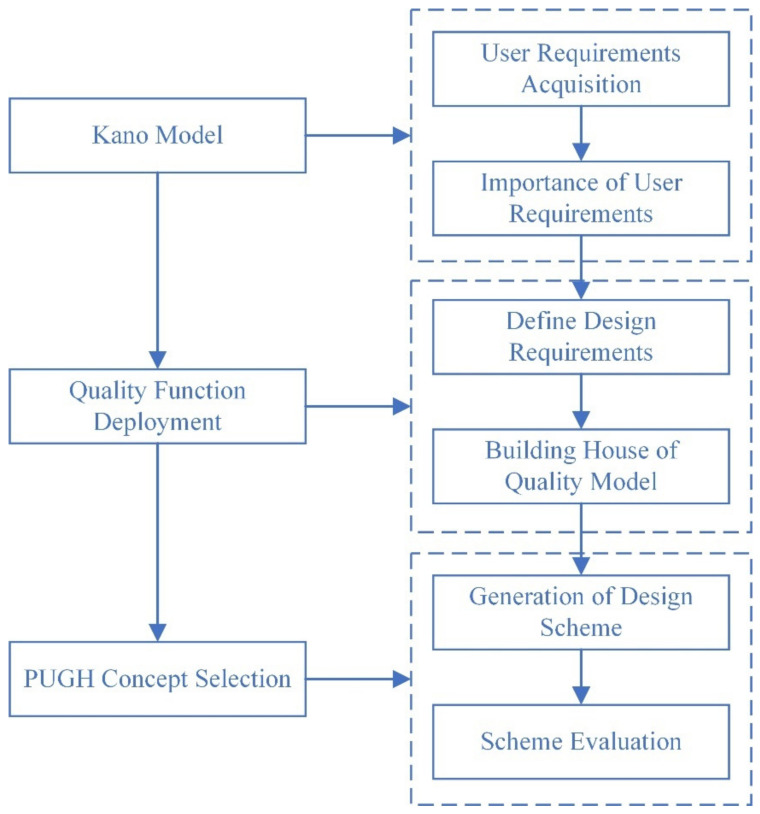
Research method framework.

The QFD-based research framework of developing a sustainable smart refrigerator design that optimizes user experience in the elderly is shown in Figure 2. First, the Kano model is used to analyze the demand information of the elderly group and determine the importance of user needs. Secondly, based on QFD, the user requirements are converted into design requirements, and the “user requirements–design requirements” house of quality is established; the correlation matrix is obtained, and the importance of the design requirements is confirmed through quantitative analysis. Finally, the design scheme is selected according to the PUGH concept, and the optional scheme is evaluated to determine the final design scheme [42,43].

### 3.1. Kano Model

#### 3.1.1. User Requirements Acquisition

In order to more deeply understand user needs and improve user experience, we need to collect user needs through interviews and then sort and integrate them, taking the higher user needs as the main needs, removing the lower needs, determining the importance of smart refrigerator functions to users, and dividing the sorted needs through the KJ (Kawakita Jiro) method, which is also known as the affinity diagram method. This is a kind of induction from and summary of the messy information method, which is mainly divided into different levels according to its use in clarifying the corresponding relationship between information and conditions [44].

#### 3.1.2. Importance of User Requirements

Based on the Kano model, user requirement questionnaires have been designed to quickly prioritize requirements via a 2-step system of positive/reverse questions and attribute type summarizations of product features/services. The user satisfaction rating in each question can be one of the following five rating levels: very satisfied, taken for granted, indifferent, barely acceptable, and dissatisfied. Although research has shown that seven rating levels capture more accurate data, five rating levels were used in this study to prevent the older adults surveyed from finding it too cumbersome, and thus losing patience. This structure is more concise and easy to understand, and it helps older adults to judge and select items easily. The results of the questionnaire survey are judged by type according to the Kano model matrix, which includes must-be needs (M), one-dimensional needs (O), attractive needs (A), indifferent needs (I), reverse needs (R), and questionable results (Q), where one-dimensional needs and attractive needs play a key role in improving user satisfaction, reverse needs are not used as reference data, and questionable results are eliminated to prevent messy data from affecting the analysis results; see Table 1 below.

Based on the Kano model, a single qualitative analysis is not enough to capture user needs, so the adjustment coefficient *k_i_* is introduced, taking the values 1.5, 1, 0.5, and 0 to indicate attractive needs, one-dimensional needs, must-be needs, and indifferent needs, respectively [45]. *w_i_* is the weight of the *i*-th indicator [46], and the final user need’s importance *w_i_* is shown in Equation (1). We use the user needs to identify pain points and problems in the experience, and through quantitative analysis more accurately identify the real needs of users, providing a reference for the subsequent design research.

(1) $$W_{i}=\frac{w_{i}k_{i}}{\sum_{i=1}^{n}w_{i}k_{i}}$$

### 3.2. Quality Function Deployment

#### 3.2.1. Define Design Requirements

Combining the user needs derived from the Kano model, design requirements that meet the needs characteristics of the elderly are proposed. The purpose of smart refrigerator design is to optimize a comfortable and efficient use process, improve the usability, ease of use and pleasure of smart refrigerators, enhance the experience of elderly people using smart refrigerators, and enhance user satisfaction.

#### 3.2.2. Building House of Quality Model

Based on QFD, a quality house model of user needs and design requirements is established, and the correlation between each part and function is expressed by a score, with 5, 3 and 1 representing high correlation, moderate correlation and weak correlation, respectively. To ensure that each user need is correlated with the design requirements, the quality house model is examined, and the core design requirements are highlighted to carry out the design using the advantages of QFD. The calculation method used to determine the importance of design requirements *H_j_* is shown in Equation (2), and *R_ij_* is the relationship degree value between the *i*-th elderly need and the *j*-th design requirement.

(2) $$H_{j}=\sum_{i=1}^{n}W_{i}R_{ij}(j=1,2,3,\ldots ,m)$$

### 3.3. PUGH Concept Selection

Through PUGH concept selection, the design scheme is evaluated according to a comprehensive score, and the best design decision and optimization direction are obtained. The results are obtained from the sum of the importance values of the evaluation. The new scheme generated by comparing the advantages and disadvantages of the scheme is added to the evaluation, and the design scheme is analyzed in detail to determine the final scheme.

#### 3.3.1. Design Solutions Generation

According to the importance of design requirements determined by the quality house model, the higher the score means, the more important are the specific requirements to improving the satisfaction of the elderly with the smart refrigerator; the identified important design requirements will be applied to the design scheme. Smart refrigerator products usually consist of product design elements such as shape, material, process, color, operation mode, practical durability, safety, and a series of visual interface elements such as menus, text, and icons. The smart refrigerator features collected include most of the product and interface styles, and the top four design requirements are ranked in terms of importance as the main elements, with the others featured as auxiliary elements.

#### 3.3.2. Program Evaluation

The concept level assessment identifies one of the schemes involved in the evaluation as the reference standard, and it indicates the difference in relative performance according to a score of 1–5 (1 means much worse than the reference standard, 5 means much better than the reference standard); the magnitude of the score is calculated by combining the importance of the design requirements, and the final score of the assessment is given in Equation (3). *v_ij_* denotes the original score of concept *j* on the *i*-th criterion [47]. The product design solutions have been ranked from highest to lowest according to the comprehensive score to derive the best design decision and the optimization direction, summarize the principles of smart refrigerator design targeting the elderly, and enhance the satisfaction of elderly users’ experience.

(3) $$V_{i}=\sum_{i=1}^{n}H_{j}v_{ij}$$

## 4. Case Study

### 4.1. Kano Model

#### 4.1.1. User Requirements Acquisition

**Figure 3:**
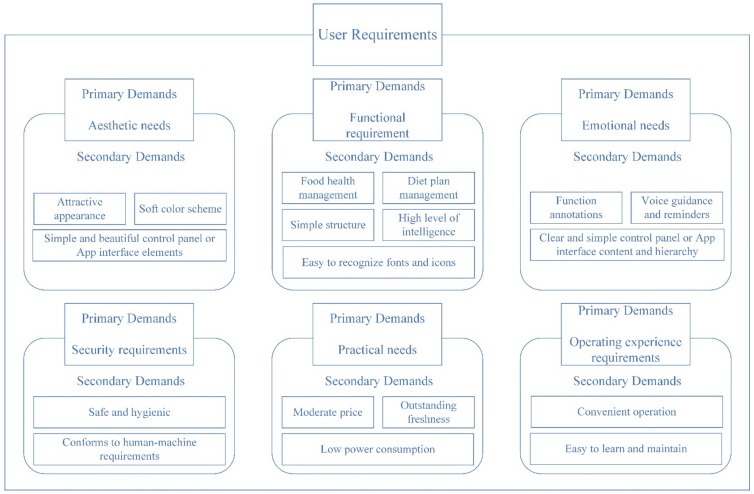
Collected user requirements.

When elderly people are over 80 years old, their cognitive function may decline rapidly, which might have an impact on the quality of smart refrigerator research data. In order to ensure that the demand information is well formulated, this study was conducted on 60 users aged between 60 and 79 years old who have experience in using smart refrigerators. To ensure the representativeness of the sample, we stratified the data collection approach by age, gender, and first- and second-tier urban areas in China, and we analyzed the data in conjunction with relevant departmental survey data. The elderly users’ original demand descriptions were obtained through interviews, and the results of this demand information show that most of the smart refrigerator users have common requirements, and a few users have different requirements. In order to avoid an unfocused design, the common requirements of most elderly people were selected to categorize the information using the KJ method, and 6 primary requirements and 18 secondary requirements were obtained—see Figure 3.

Safety needs relate to whether the product is safe and hygienic, in line with the human–machine interface aspects, and help to enhance the safety of the elderly when using the product. Practical needs concern whether the product has low power consumption, moderate price, etc. Operating experience refers to the need to provide specific operation information for the elderly, with convenient operation reducing the cognitive burden of elderly users, and improving the user’s operating efficiency.

#### 4.1.2. Importance of User Needs

A combination of electronic and paper-based questionnaires was used. To ensure the representativeness of the sample, convenience sampling method was used in two cities, Shanghai and Nanjing, to survey users aged between 60 and 79 years old with experience in using smart refrigerators, focusing on areas with a dense elderly population, such as residential areas, sports parks, and square fitness areas in the main urban areas. The two cities were selected because they represent the first- and second-tier cities in China and have a more developed economy, relatively high income and living standards of the elderly, and a thriving consumer market for smart kitchen appliances. A total of 60 questionnaires were distributed, and respondents spent no less than 300 s answering them, of which 59 results were valid, representing a return rate of 98.3%. The collected questionnaires were quantitatively analyzed according to formula (1), and the attractive demands were identified as C_11_ beautiful shape, C_12_ soft color scheme, C_21_ food health management, C_22_ diet plan management, C_33_ voice guidance and reminder and C_53_ moderate price. The one-dimensional demands were C_13_ control panel or app interface elements being simple and beautiful, C_41_ safety and hygiene, C_42_ meeting human–machine requirements, C_51_ low power consumption, C_52_ outstanding freshness, C_61_ convenient operation, C_62_ easy to learn and maintain, C_23_ easy recognition of fonts and icons, C_24_ high degree of intelligence and C_31_ clear control panel or app interface content and simple hierarchy. The indifferent requirements are C_25_ simple structure, C_32_ functional notes, and no reverse requirements. According to the Kano model, the indifferent requirements of C_25_ simple structure and C_32_ functional annotations were removed. The original weights were obtained according to a survey of the importance of the needs of the elderly, and the final importance values of the user needs are shown in Table 2.

### 4.2. Quality Function Deployment

#### 4.2.1. Define Design Requirements

The user requirements analyzed according to the Kano model were transformed into design requirements by QFD, and 15 product designers were invited to refine the design requirements according to their professional knowledge. The design features with 70% common recognition by experts were retained, while the remaining design features were deleted to simplify the design analysis process. Finally, these user requirements were converted into design requirements for detailed implementation (see Table 3). For example, the first level design requirement, “D_1_ visual design”, includes a series of design requirements for product display and data display, such as material, process and color, shape, interface color scheme, etc. Meanwhile, the requirement “D_2_ functional system design” refers to the need to meet the diversified needs of the elderly and help them make effective decisions. The first-level design requirements are “D_11_ CMF design”, “D_12_ modeling design”, “D_13_ interface color scheme design”, “D_21_ multi-functional personalized design”, and “D_12_ color scheme design”. “D_21_ multi-functional personalization design”, “D_22_ interface text design”, “D_23_ interface graphic icon design”, etc., are aimed at improving the recognition of the product and interface display, the pleasure of using and the enjoyment of beauty, in order to facilitate operation and enhance the user experience.

#### 4.2.2. Build a Quality House Model

The “left wall” and the “ceiling” of the quality house convey the needs of elderly users and the design requirements, respectively (Table 2 and Table 3). The correlation between each group of user needs and design requirements is expressed as a score, with a strong correlation represented by 5 points, a medium correlation by 3 points, a weak correlation by 1 point, and no correlation having no score. The scores were based on a scoring panel of three elderly users with two or more years of experience using smart refrigerators, and interactive interface and industrial design experts, as shown in Table 4.

### 4.3. PUGH Concept Selection

In this study, the PUGH concept was selected to grade the resulting design solutions, and the optimal design solution was determined by the score, in order to achieve a satisfactory user experience.

#### 4.3.1. Design Solutions Generation

**Figure 4:**
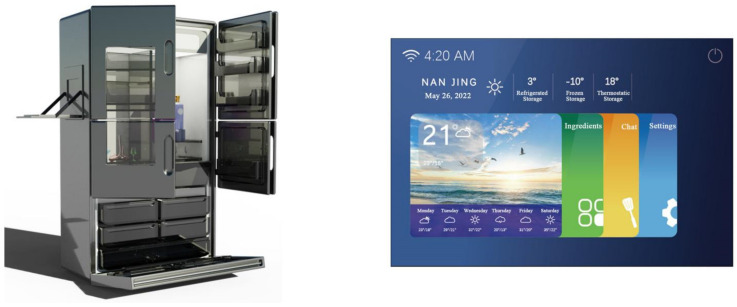
Scheme (A): Product and interaction interface.

**Figure 5:**
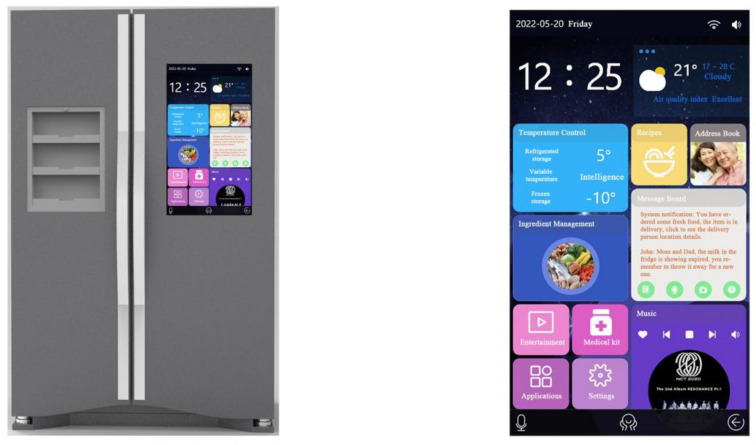
Scheme (B): Product and interaction interface.

**Figure 6:**
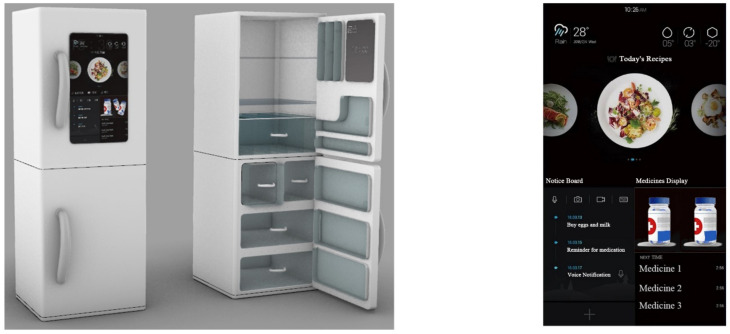
Scheme (C): Product and interaction interface.

**Figure 7:**
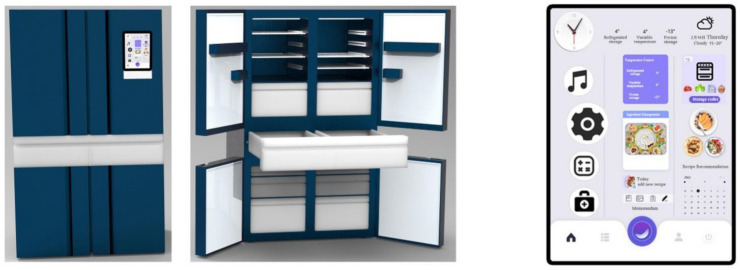
Scheme (D): Product and interaction interface.

According to the preliminary quality house matrix analysis, the design requirements that are of highest importance and that can be used in the design program are “D_21_ multi-functional personalized design”, “D_41_ human–machine design”, “D_63_ manual touch screen design “, “D_23_ icon graphic design of the interface”, “D_31_ voice control design”, “D_33_ functional classification design of the interface”, and “D_34_ icon and functional module design”. “D_34_ layout design of icons and functional modules”, “D_12_ styling design”, “D_11_ CMF design”, “D_32_ interface navigation design”, and “D_62_ modular design” were used as the main references for the design of the smart refrigerator, and the four solutions were evaluated—see Figure 4, Figure 5, Figure 6 and Figure 7.

Scheme (A)—First, each floor of the refrigerator is divided into six partitions, and each partition holds the same or similar items, making it more convenient for the user to regulate the refrigeration and freshness of each item, and making the fridge itself more resistant to viruses resulting from food mixing. The side of the refrigerator also features a temporary shelf for items. Secondly, the interface is designed to be user-friendly so that seniors can manage ingredients, view videos, chat, etc. At the same time, this refrigerator can also interface with the cell phone terminal; children can speak to the elderly and messages can be displayed.

Scheme (B)—This is a smart refrigerator designed for the elderly. It is different from the ordinary refrigerator products on the market; it is concerned with care for the elderly, with consideration of leg and foot inconvenience and inconvenient bending via the foot touch switch placed below; it features a large intelligent display, and a simple and easy-to-operate visualization interface, ensuring good operability and enhancing the life quality of the elderly via intelligent assistance.

Scheme (C)—This smart refrigerator for the elderly takes into consideration the height of the user for easy access, and has an exclusive storage space for medications. The refrigerator connects to a cell phone to control the dosage and time of medications. Each medicine box has a corresponding chip to identify the medicine, which prevents the elderly user from taking the wrong medication, avoids the secondary pollution of medication, and effectively guards the health of the elderly.

Scheme (D)—This is a smart refrigerator designed for the elderly. It is designed according to the height and physique of the user, and the storage space is partitioned to meet the needs related to medicine and temperature control; it is understood that the eyesight of the elderly will decline, and so a calm dark blue is chosen as the color of the product. Frozen food can be identified by photo code, making it easy to find, and there can be intelligent voice prompts.

#### 4.3.2. Schemes Evaluation

The product and interaction interface were evaluated according to the design requirements, and Scheme C was used as the final reference concept. To ensure the objectivity of the evaluation, the concepts were scored using Equation (3) to confirm whether the design requirements were reasonable, and the total score and importance of each solution were obtained based on the scores. This enabled us to arrive at the best solution, and the results are shown in Table 5.

## 5. Discussion

To respond to the serious threat posed by the pandemic, as well as the goal of achieving sustainability of health and well-being in the elderly and achieving sustainable industrialization and innovation, we have attempted to improve the existing smart refrigerator design for the elderly using a systematic approach combining Kano–QFD–PUGH to enhance user experience and thus achieve the rapid development of smart home products. In the case study, we have demonstrated the effectiveness of the integrated Kano–QFD–PUGH approach in addressing user needs, design requirements, design solutions and their internal dependencies. These results help to form further design strategies for the design of smart refrigerators for the elderly.

Via Kano–QFD–PUGH, six user needs were identified, namely, esthetic needs, functional needs, emotional needs, safety needs, practical needs, and operational experience needs. The scoring of the importance of user needs by older users shows that functional needs are the most critical. Most people tend to focus on whether the smart refrigerator can provide safety, health and ease of use. Therefore, we should first consider how to meet these needs and combine design solutions to propose strategies based on them. Second most important are the practical needs. As the smart refrigerator is the most commonly used product in the daily home life of the elderly, its practicality is aimed at the elderly, with the specific needs of low power consumption, outstanding freshness and moderate price; these are concerns because the elderly may live more frugally compared to young people, being more concerned about price and power consumption, in order to achieve food preservation at a lower cost. At the same time, we cannot ignore the esthetic needs, such as smart refrigerator volume and space consumption. The smart refrigerator product is defined by its shape, color, material, texture, information interface and other elements; through the design of the communication platform in the product, communication can come to play a key role in the practical value and meaning of the design. Secondly, safety is also a concern of the elderly, but elderly users scored the safety demand low; it is not that this demand is not important, but rather that the functionality and safety complement each other, and if reasonable functionality is achieved, then the safety will also be ensured [48]. Functional needs are the most critical for elderly users, and also satisfy the safety needs of elderly users to a certain extent. Again, in terms of the operation experience requirements of elderly users, when facing the complex design of smart refrigerators and the accompanying new operation experience, the unprepared will feel unfamiliar with the product, and be unable to use it. Therefore, we optimize the smart refrigerator design operation and thus improve the operation experience by designing a product that meets the standards of the elderly, which is not only convenient for the user, but is also easy to promote, thus expanding the market prospects of the product. Last are the emotional needs of the elderly; although this requirement has the least weight among all needs, it still plays an important role in relation to the life and health of the elderly, and their happiness in life. According to Maslow’s hierarchy of needs theory [49], when people’s basic physiological needs and security needs are satisfied, they will pursue higher-level needs, including emotional needs.

Six design requirements are put forward. With the constant growth of technology, the demands of the elderly for smart refrigerators are also changing in terms of functional system design. In addition to meeting the storage and freshness demands of the elderly, smart refrigerators address other requirements, such as medical care, health management, intelligent features, and recreational and entertainment needs. As the kitchen is a significant location in the lives of the elderly, the disadvantages of conventional refrigerators restrict the fulfilment of their demands. Intelligent refrigerators may meet the demands not met by conventional refrigerators by including intelligent functionality. The functional design needs of intelligent refrigerators expand and improve through time, and so does the life satisfaction of the elderly. Product intelligence is mostly evident in the utilization of big data to provide customers with more convenient home life services [50]. Due to the gap in age and experience between the old and the young, the elderly often choose to purchase practical things. Therefore, smart refrigerators must not only offer technological support and great performance, but they must also address the pain spots and demand points of customers throughout product use, and seek breakthroughs to address the issues. For instance, senior consumers tend to be thriftier than their younger counterparts, preferring items with features of reasonable costs, minimal power consumption, and excellent preservation. Therefore, aging-friendly design is an intrinsic need in product development. The only items that may really captivate the elderly are those that cater to their routines and provide significant improvements to their life. Thus, they may become an integral part of their life, and even lead people to make sacrifices for the said items. In terms of visual design, as smart goods continue to develop from the standpoint of product appearance, a delicate and beautiful appearance becomes more important to customers. The majority of people feel that the elderly do not have a sense of esthetics and do not respect the look of a product. As a result, the majority of smart goods on the senior market do not consider the beauty of the product from the elderly person’s viewpoint. However, as technology evolves, so does the quality of eyesight support for the elderly, who thus demand goods that are both esthetically pleasing and provide a pleasant experience. Therefore, the appearance and size of the product should conform to the ergonomics of specific elderly groups, and have a certain fault tolerance rate, making it truly designed for the elderly [51]. From the perspective of the interactive interface, the function of the smart refrigerator interface for elderly users is to provide effective information to elderly users in a timely manner, such that users can extract information more effectively. Using levels to differentiate interface information, the layout and color scheme should be complementary. Considering the capacity of older users to extract crucial information from text based on their personal characteristics, such as education and profession, as well as their ability to identify icons, the product should not employ icons that will cause confusion among elderly users. The design should consider the psychological features of senior consumers in order to actively lessen their visual load. In addition, it is important to consider the physiological and psychological characteristics of the older user. When the elderly use a smart refrigerator, they may experience eye pain owing to the screen’s brightness, and long-term usage will cause eyestrain. Despite the fact that yellow, red, and other hues may improve the interface’s recognition, long-term viewing will produce visual fatigue due to the more stimulating nature of the wavelength. With the aim of guaranteeing comfort, the design should promote the recognition of the interface [52]. In conclusion, visual design is a main priority for designers, not only regarding the age-appropriateness of the design, but also in relation to the interface’s color scheme, brightness, and typeface. In terms of safety design, safety is a necessary principle in the design of elderly smart refrigerator products, as the elderly group, due to the decline of physical functions, lacks in physical fitness, and some of their functions will have begun to deteriorate, making them prone to unsteadiness and falling. To minimize physiological harm, the design of smart refrigerators for the elderly must be safe and trustworthy from the human–machine and structural perspectives [53]. In terms of emotional and experiential design, the smart refrigerator must be supportive of the emotional experience and psychological feelings of the elderly when they interact with it, considering comfort, pleasure, and accomplishment, which can be encouraged via voice control and output, interface navigation, the functional classification of the interface, the layout of icons and functional modules, the semantics of the interface, and so on. When the elderly and the smart refrigerator form a good interactive relationship, the elderly will experience comfort, which leads to a sense of trust, and through the continuous accumulation of trust, not only will the elderly feel pleasure and satisfaction in the use of the smart refrigerator, but it will also increase the elderly consumers’ desire to purchase, thereby improving the market [54].

## 6. Sustainable User Experience Optimization Design Strategies for Smart Refrigerators for the Elderly

According to the relative weights and internal relationships of user needs and design requirements, combined with the characteristics of the elderly, this paper puts forward a design strategy of an intelligent refrigerator user experience optimization design, considering elderly users, which guides the design via five aspects—shape, function, emotion, interface layout and use—in order to alleviate the impact of the epidemic on the physiological and psychological health of the elderly. It is worth noting that the link between the design strategy and user needs and design requirements proves the importance of these aspects. At the same time, according to the early house of quality matrix analysis, the design requirements of “D_21_ multi-function personalized design”, “D_41_ man–machine design”, “D_63_ manual touch screen design”, “D_23_ interface icon graphic design”, “D_31_ voice control design”, “D_33_ interface function classification design”, “D_34_ icon and function module layout design”, “D_12_ modeling design” and “D_11_ CMF design” are of high importance, and can be used in the design scheme. As the main design requirements, “D_32_ interface navigation design” and “D_62_ modular design” propose corresponding design strategies for smart refrigerators. Therefore, the design strategy can be summarized as follows.

### 6.1. Morphological Design Strategy

The design strategy combines “D_12_ shape design” and “D_11_ CMF design”. The smart refrigerator is a product that is often used in the daily life of the elderly, and so its form should be robust, generous, friendly-feeling, soft and affable, which will impart a pleasant feeling to the elderly. The color contrast should not be too strong, and it is appropriate to use brighter intermediate colors; accessories can use more pure colors, with decorative embellishments. In addition, through the product’s shape, color, and other aspects of morphological design, it is possible to stimulate and motivate the elderly; this is beneficial to maintaining a healthy psychological state and stimulating the emotional experience of the elderly.

### 6.2. Functional Design Strategy

This design strategy comprises “D_21_ multi-functional personalized design” and “D_62_ modular design”, and concerns the psychological characteristics of the elderly. It is known that the elderly pay attention to practical value, and are sensitive to the performance–price ratio of products, hoping to buy practical products at affordable prices. Therefore, in the design of products for the elderly, we should focus on the practicality of product functions, which is mainly reflected in the multi-functionalization of simple products, the simplification of complex products, and a reduction in unnecessary functions of products. To adapt to the complex and diverse health and safety needs of the elderly, “D_62_ modular design” separates fresh storage from the freezer, and controls both with air-cooling and compression refrigeration systems, respectively. This achieves structural optimization as well as energy saving. As well as taking into account the placement of certain food products, the original push–pull structure is changed, and rotating trays are used instead of drawers and panels to facilitate use and cleaning. This design strategy seeks the best balance between function and price, aiming at designing good-quality and inexpensive products that will improve the ease of use of the products for the elderly; this demands good communication, such that learning to use the products is not only easy, but the products can meet their physical and emotional needs.

### 6.3. Emotional Design Strategies

This design strategy is related to “D_23_ icon graphic design of interface”. Based on the consideration of the living conditions, cultural background and psychological characteristics of the elderly, we pay attention to the emotional resonance between the product and the elderly users. We define the interface design elements based on the esthetic preferences and emotional needs of the elderly, and we strive to bring a sense of closeness to the elderly both visually and through tactile experience. Therefore, we adopt simple icons to reduce the memory burden of elderly users, and we avoid unnecessary operations. Considering the potential negative emotions of the elderly, a simple color composition is used to help them to complete their tasks effectively and thus establish emotional confidence, thus improving the experience of using the product. The logic of the product is in line with the cognitive rules well established in the elderly, so as to reduce the distance of the elderly from the smart home product and the possibility of their rejecting it, and to increase the sense of closeness and dependence.

### 6.4. Interface Layout Design Strategy

The design strategy is related to “D_34_ icon and function module layout design”, “D_32_ interface navigation design” and “D_33_ interface function classification design”. The card-type layout makes the interface neat, reasonable and smooth, such that the line of sight can be easily integrated. The flat icons are simple and clear, and the functions are obvious, which accommodates the operation habits and cognitive patterns of the elderly, improving the operation efficiency, reducing the time spent on extra functions, helping the user overcome cognitive barriers and carry out human–computer dialogue more easily, guaranteeing the use experience, and improving satisfaction.

### 6.5. Use-Level of Design Strategies

This design strategy involves “D_63_ manual touch screen design”, “D_31_ voice control design” and “D_41_ human–machine design”. As the elderly age, their physical structure, senses, intelligence, receptiveness and ability to adapt to the environment will deteriorate, decline, and slow down to varying degrees. Therefore, manual input and voice control as the main operation method can increase the simplicity and ease of operation of the product. At the same time, in order to ensure the safety of the elderly using the smart refrigerator, we also need to consider whether the size, physical characteristics, and dynamic and static force that must be applied are suitable, thus also meeting the physical and emotional needs of the elderly.

## 7. Implications and Limitations of the Study

The findings of this study are beneficial on multiple levels. For practitioners, the design strategies developed in this study can guide them in enhancing the satisfaction of older users with smart home products, and they reduce the physiological and psychological impacts of the epidemic on the elderly in order to promote healthy and sustainable designs for older adults. This study provides design educators and researchers with a more comprehensive understanding of user expectations and product characteristics, in order to help them in applying this study’s methodology. Finally, for government agencies, policy-makers, and community managers, this study demonstrates how designs can be refined to provide services for older adults, enhance their quality of life, and promote socially harmonious and sustainable development.

However, there are still some limitations to our study. First, the scope of this study is limited to the elderly population in densely populated communities in first- and second-tier cities in China. This is a problem because each country may comprise a wide variety of demographics, living environments, cultures, customs, lifestyles, and economies. Second, government agencies and policy-makers should consider their own conditions and circumstances when providing services to older adults. For example, smart technology, a usage requirement, should play a central role in the design of smart home products for the elderly in order to enhance their well-being. However, smart technologies are related to the construction of urban infrastructure and facilities. These expensive technologies may not be applicable to all countries’ contexts. In this case, we recommend that government agencies and policy-makers facilitate the provision of cheaper home products that can be used in the daily lives of the elderly. Finally, the weightings of requirements related to sustainability and design, such as low power consumption and corresponding power design aspects, are relatively lower than other dimensions, which are not really explored in our study. However, we cannot ignore the critical impact of these other requirements. As mentioned above, product sustainability is always a topic of concern. In this paper, we found that older adults are not as concerned with sustainability because of the long hours of labor required to keep a household running, and their established personal habits [55].

## 8. Conclusions

Within the context of the COVID-19 pandemic, the use of smart home products by the elderly can help them reduce their loneliness, improve their physical health and increase their independence. Among these, the improvement of user experience in relation to smart home products is particularly important. Therefore, this study proposes a QFD-based research method for optimizing the user experience design of smart home products for the elderly, taking smart refrigerators as an example to improve upon the designs of other products typically used. The Kano model is used to determine user requirements, and it is concluded that esthetic requirements, functional requirements, emotional requirements, safety requirements, practical requirements, and operational experience requirements are the most important for the elderly. QFD is used to transform the main requirement elements into design requirements that can improve user satisfaction. Among these, the design requirements of the highest importance include multi-functional personalized design, human–machine design, manual touch screen design, the icon graphic design of the interface, voice control and output design, the functional classification design of the interface, the layout design of icons and functional modules, styling design, CMF design, interface navigation design, and modular design. At the same time, we employ PUGH concept selection to verify the rationality of the design scheme, determine the final design scheme, and summarize the design strategy. The whole design process takes the elderly as the research object. Through qualitative and quantitative analyses, we can classify the needs and design requirements of the elderly, as well as incorporating special care for the elderly into the design of smart refrigerators.

By combining the Kano model, QFD and PUGH concept selection, the user experience optimization of smart refrigerators design for the elderly is studied, which not only provides a theoretical basis for designers to determine the most important requirements, but also gives targeted design solutions, and proposes corresponding sustainable user experience optimization design strategies. This study verifies that the research framework can be effectively applied to the user experience optimization of smart refrigerator design for the elderly, and it is also applicable to the research topic of user experience optimization of smart home products for the elderly.

Future research work will utilize a large amount of customer data to improve the quantitative analysis of the Kano model, in order to more objectively determine the functionality of the demand relationship. In addition, we will continue to improve the testing of smart home products, and conduct more case studies to verify the use of the design solution to improving the operational experience of the elderly in their actual use of smart home products, whilst also improving the applicability and feasibility of the design solution.

## Figures and Tables

**Table 1:** Judgment matrix of the user demand Kano model.

User Requirements	Reverse Questions					
Positive Questions		Very Satisfied	Right and Proper	Indifferent	Reluctantly Accepted	Dissatisfaction
	Very satisfied	Q	A	A	A	O
	Right and proper	R	I	I	I	M
	Indifferent	R	I	I	I	M
	Reluctantly accepted	R	I	I	I	M
	Dissatisfaction	R	R	R	R	Q

**Table 2:** List of importance of user requirements.

User Requirements	A	M	O	I	R	Demand Type	Original Weights	Adjustment Factor	Adjusted Proportion	Weights
C_11_ Beautiful appearance	22	10	8	19	0	A	0.053	1.5	0.080	0.082
C_12_ Soft color matching	19	9	15	11	0	A	0.053	1.5	0.080	0.082
C_13_ Simple and beautiful control panel or app interface elements	12	13	26	8	0	O	0.058	1	0.058	0.059
C_21_ Food health management	27	2	14	14	0	A	0.054	1.5	0.081	0.083
C_22_ Diet plan management	31	1	5	21	0	A	0.051	1.5	0.077	0.079
C_23_ Easy to recognize fonts and icons	11	18	15	15	0	M	0.055	0.5	0.028	0.029
C_24_ High level of intelligence	14	21	12	11	1	M	0.052	0.5	0.026	0.027
C_25_ Simple structure	12	9	6	27	3	I	0.049	0	-	-
C_31_ Clear and simple control panel or app interface content and hierarchy	11	23	17	7	0	M	0.057	0.5	0.029	0.030
C_32_ Function notes	14	11	12	22	0	I	0.054	0	-	-
C_33_ Voice guidance and reminders	23	4	11	21	0	A	0.050	1.5	0.075	0.077
C_41_ Safety and health	1	16	40	2	0	O	0.062	1	0.062	0.064
C_42_ Compliant with human–machine requirements	8	11	26	13	0	O	0.058	1	0.058	0.059
C_51_ Low power consumption	12	11	33	3	0	O	0.059	1	0.059	0.061
C_52_ Outstanding freshness	12	12	29	5	0	O	0.061	1	0.061	0.063
C_53_ Moderate price	18	10	15	15	1	A	0.054	1.5	0.081	0.083
C_61_ Easy to operate	8	11	34	6	0	O	0.061	1	0.061	0.063
C_62_ Easy to learn and easy to maintain	13	7	30	8	0	O	0.058	1	0.058	0.059

**Table 3:** Design requirements.

First-Level Design Requirements	Second-Level Design Requirements
D_1_ Visual design	D_11_ CMF Design (Color; Material; Finishing) D_12_ Modeling design D_13_ Color scheme of the interface D_14_ Layout design of the interface D_15_ Brightness design of the interface
D_2_ Functional system design	D_21_ Multi-functional personalized design D_22_ Text design of the interface D_23_ Icon graphic design of the interface
D_3_ Emotional design	D_31_ Voice control design D_32_ Interface navigation design D_33_ Functional classification design of the interface D_34_ Layout design of icons and functional modules
D_4_ Safety design	D_41_ Human–machine design D_42_ Structural design
D_5_ Practical design	D_51_ Power design D_52_ Inverter technology application D_53_ Overall volume
D_6_ Experience design	D_61_ Semantic design of the interface D_62_ Modular design D_63_ Manual touch screen design

**Table 4:** User requirements and design requirements house of quality.

		Level of Importance	D_1_					D_2_			D_3_				D_4_		D_5_			D_6_		
			D_11_	D_12_	D_13_	D_14_	D_15_	D_21_	D_22_	D_23_	D_31_	D_32_	D_33_	D_34_	D_41_	D_42_	D_51_	D_52_	D_53_	D_61_	D_62_	D_63_
C_1_	C_11_	0.082	3	5											1				3			
	C_12_	0.082	5		5																	
	C_13_	0.059			5	3	3		1	5		5		5								5
C_2_	C_21_	0.083						5			1		5							1		5
	C_22_	0.079						5			1		5							1		5
	C_23_	0.029					5		5	5		1		5						5		1
	C_24_	0.027	1	1				5			5				1		3	3		1	3	3
C_3_	C_31_	0.030				3	5		1	5		5	5	5						5		3
	C_33_	0.077						5			5				1					1		
C_4_	C_41_	0.064	1	1											5	3	3	3			5	
	C_42_	0.059	3	3	3	1	5		3	5	3	5		5	5	3	3			5		
C_5_	C_51_	0.061													3		5	5	1			
	C_52_	0.063													3	3	3	5			3	
	C_53_	0.083	3	5				5							3		3	5	3		1	
C_6_	C_61_	0.063	1	3	3	3	5	5	5	5	5	5	5	3	5	3	1		3	3	3	5
	C_62_	0.059	1	1	1	3	1	5	5	5	5	3	3	5	5	1			1	5	5	5
	Level of importance		1.295	1.341	1.130	0.692	1.141	2.355	1.021	1.495	1.469	1.261	1.452	1.369	2.032	0.806	1.256	1.308	0.804	1.340	1.157	1.915
	Order of Importance		11	8	16	20	15	1	17	4	5	12	6	7	2	18	13	10	19	9	14	3

**Table 5:** Correlation between user needs and evaluation elements.

Design Requirements	Level of Importance	Alternative Schemes			
		A	B	C	D
D_11_ CMF Design	1.295	4	3	3	4
D_12_ Modeling design	1.341	4	3	3	4
D_13_ Color scheme of the interface	1.130	5	3	2	5
D_14_ Layout design of the interface	0.692	4	3	2	4
D_15_ Brightness design of the interface	1.141	4	2	2	4
D_21_ Multi-functional personalized design	2.355	5	4	4	5
D_22_ Text design of the interface	1.021	4	3	3	4
D_23_ Icon graphic design of the interface	1.495	4	3	2	4
D_31_ Voice control design	1.469	4	4	3	4
D_32_ Interface navigation design	1.261	4	4	2	3
D_33_ Functional classification design of the interface	1.452	4	4	3	4
D_34_ Layout design of icons and functional modules	1.369	4	3	3	4
D_41_ Human–machine design	2.032	5	3	3	4
D_42_ Structural design	0.806	5	3	3	4
D_51_ Power design	1.256	5	5	5	5
D_52_ Inverter technology application	1.308	5	5	5	5
D_53_ Overall volume	0.804	4	4	3	4
D_61_ Semantic design of the interface	1.340	4	4	3	4
D_62_ Modular design	1.157	4	3	3	4
D_63_ Manual touch screen design	1.915	5	5	5	5
Total Score		117.358	96.415	85.511	113.259

The evaluation results show that Option A has the highest score, of 117.358, followed by Option D with 113.259, Option B with 96.415, and the lowest is Option C with 85.511. The best design solution can be selected and used for product development via this scoring approach.

## Data Availability

Not applicable.
